# Essential role of calcium in extending RTX adhesins to their target

**DOI:** 10.1016/j.yjsbx.2020.100036

**Published:** 2020-09-08

**Authors:** Tyler D.R. Vance, Qilu Ye, Brigid Conroy, Peter L. Davies

**Affiliations:** Department of Biomedical and Molecular Science, Queen’s University Kingston ON, Canada

**Keywords:** Calcium coordination, Bacterial adhesin, Adhesion protein, Beta-sandwich domains, *Mh*Lap, *Marinobacter hydrocarbonoclasticus* long adhesion protein, *Ah*Lap, *Aeromonas hydrophila* long adhesion protein, *Mp*IBP, *Marinomonas primoryensis* ice-binding protein, RTX, Repeats in toxin, vWFA, von Willebrand Factor A

## Abstract

RTX adhesins are long, multi-domain proteins present on the outer membrane of many Gram-negative bacteria. From this vantage point, adhesins use their distal ligand-binding domains for surface attachment leading to biofilm formation. To expand the reach of the ligand-binding domains, RTX adhesins maintain a central extender region of multiple tandem repeats, which makes up most of the proteins’ large molecular weight. Alignments of the 10-15-kDa extender domains show low sequence identity between adhesins. Here we have produced and structurally characterized protein constructs of four tandem repeats (tetra-tandemers) from two different RTX adhesins. In comparing the tetra-tandemers to each other and already solved structures from *Marinomonas primoryensis* and *Salmonella enterica*, the extender domains fold as diverse beta-sandwich structures with widely differing calcium contents. However, all the tetra-tandemers have at least one calcium ion coordinated in the linker region between beta-sandwich domains whose role appears to be the rigidification of the extender region to help the adhesin extend its reach.

## Highlights

• Elongated beta-sandwich repeats are a major part of bacterial RTX adhesins.
• The repeats are arranged in tandem to extend away from the bacterial surface.
• Calcium ions are coordinated in the linkers between repeats to stiffen the protein.
• Rigidification of the tandem repeats further helps extension of the adhesin.
• The repeats differ greatly between species, but all have Ca^2+^ in their linkers.

## Graphical abstract

**Figure:**
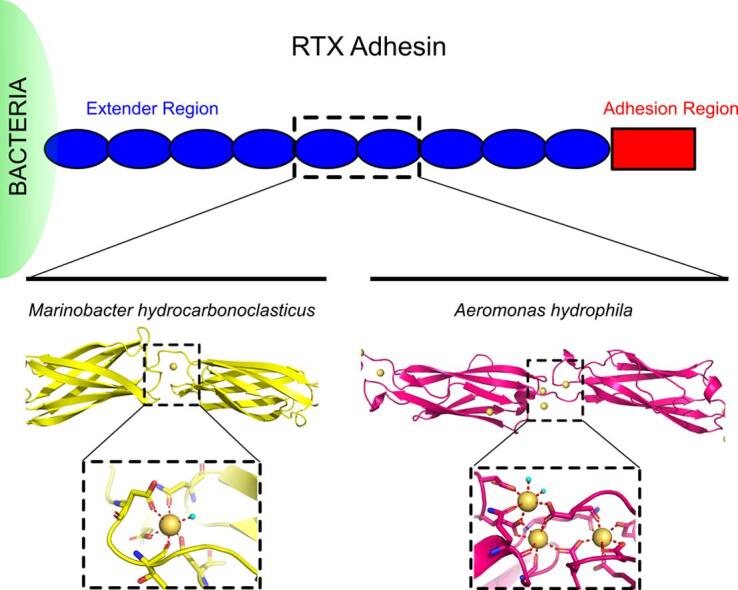

## 1 Introduction:

The beta sandwich is a ubiquitous protein fold. In its most basic form, the domain consists of at least two antiparallel beta sheets, the amphipathic nature of which leads to a hydrophobic core that holds the secondary-structure elements together (Chothia and Janin, 1981, Cohen et al., 1981). While additional strands or alpha helices are often present, these elements are not a prerequisite for all beta sandwiches. Furthermore, the number of strands that make up each beta sheet can differ between families (Chothia et al., 1997), leading to a diverse collection of domains that – though all part of the sandwich-like proteins group – maintain a variety of shapes and sizes.

There are at least 69 protein superfamilies that fall under the sandwich-like protein umbrella (Kister et al., 2002), with many of the best known being named after the heavily studied eukaryotic forerunners of the group. These include immunoglobulin-like, fibronectin III-like, and cadherin-like families (Bork et al., 1994, Leahy et al., 1992, Shapiro et al., 1995). Interestingly, the titular representatives for all these families are extracellular proteins that take part in ligand recognition and/or cellular adhesion (Singh et al., 2010, Takeichi, 1990, Williams and Barclay, 1988). Indeed, beta-sandwich domains appear well suited to these types of protein, being incorporated into extracellular proteins in both eukaryotes and prokaryotes with remarkable frequency. For example, regions of tandem beta-sandwich repeats are a hallmark of many adhesion proteins: a group of large, repetitive proteins that facilitate contacts between cells and their environment (Klemm and Schembri, 2000). These regions are found in both multi-cellular organisms (ex. integrin and cadherin, which maintain contacts between cells and extracellular matrix components (Campbell and Humphries, 2011, Takeichi, 1990)), and in single-celled organisms, where adhesion proteins allow bacteria to stick to surfaces (Guo et al., 2012, Ivanov et al., 2012, Leo et al., 2010, Schilling et al., 2001, Wagner et al., 2014) and cluster together to make bacterial communities known as biofilms (Borlee et al., 2010, Dong et al., 2020, Kikuchi et al., 2005, Martínez-Gil et al., 2010).

In all cases, the purpose of the tandem beta-sandwich domains appears to be the provision of an extended reach that projects the ligand-recognizing domains of the protein away from the cell surface (Gerlach et al., 2008, Guo et al., 2012). The beta-sandwich fold is well adapted to this role. The domain’s N and C termini are oriented opposite of each other, allowing tandem repeats to proceed in a linear fashion. Beta-sandwich domains are often exceptionally strong, maintaining their fold under intense strain; beta sandwiches from adhesion proteins, cohesins, and the muscle protein titin are able to remain folded when subjected to forces above 300 pN (Li et al., 2002, Lu et al., 1998, Oude Vrielink et al., 2017, Valbuena et al., 2009). Additionally, many beta sandwiches augment their fold or stability through the coordination of divalent cations, which are rare within but abundant outside the cell. Cadherins are known to bind calcium in the otherwise flexible loop regions between beta-sandwich domains, thereby rigidifying the structure and facilitating the homotypic interactions between cadherin molecules (Boggon et al., 2002, Kim et al., 2011, Koch et al., 1997). A similar calcium-dependent strategy has been recently discovered in bacterial adhesion proteins (adhesins). The extender beta sandwiches from an epithelial adhesin, SiiE, produced by *Salmonella enterica* require calcium for thermal stability and resistance to proteolysis (Peters et al., 2017), while the massive ice-binding adhesion protein from the Antarctic bacterium *Marinomonas primoryensis* completely loses its tertiary structure in the absence of calcium (Guo et al., 2013). Like cadherin, structure determination of small segments from both proteins showed the presence of calcium ions that coordinate to the linkers between beta-sandwich domains (Griessl et al., 2013, Vance et al., 2014), thereby producing a rod-like structure crucial for proper adhesion.

SiiE and the *M. primoryensis* ice-binding protein (*Mp*IBP) belong to the same family of adhesion proteins, known as the RTX adhesins, which are widespread amongst Gram-negative bacteria (Guo et al., 2019a, Guo et al., 2019b, Satchell, 2011). RTX adhesins have been implicated in a variety of bacterial survival strategies, including pathogen infection (Cirillo et al., 2001, Syed et al., 2009, Wagner et al., 2014), symbiotic colonization (Hinsa et al., 2003, Martínez-Gil et al., 2010), and microbial community development (Guo et al., 2017). All known examples maintain some number of beta sandwiches for extension, though the exact number of repeats, protein family in which they reside, and sequence identity vary drastically between proteins (Guo et al., 2019a, Guo et al., 2019b). While it is assumed that all these regions require calcium, only the two aforementioned examples have been structurally characterized.

In this study, we analyzed extender regions from two additional RTX adhesins, including the long adhesion proteins from *Marinobacter hydrocarbonoclasticus* (*Mh*Lap) – an oil-eating marine bacterium – and *Aeromonas hydrophila* (*Ah*Lap) – an opportunistic pathogen of fish and mammals. From these proteins, constructs comprised of four beta-sandwich repeats, called tetra-tandemers, were produced and subjected to structural determination and biophysical characterization. Here we show that while the sequences, structures, and calcium requirements of these domains differ widely, the strategy of using calcium for rigidification of the linker between domains is consistent.

## 2 Materials and methods

### 2.1 Molecular cloning, protein expression and purification

To produce the tetra-tandemers for *Mh*Lap (NCBI: WP_014422746) and *Ah*Lap (NCBI: WP_011707240), genes encoding four of the beta-sandwich repeats in tandem were synthesized by GeneArt (Thermofisher). As previously described, codon optimization for expression in *Escherichia coli* was balanced with differentiating the repeats using codon degeneracy (Vance et al., 2014). These genes (Fig. S1) were then ligated into pET28a vectors using *Nde*I and *Xho*I restriction cut sites, thereby encoding an N-terminal His6-tag, and transformed in Top10 competent *E. coli* cells (Thermofisher) for plasmid amplification. Clones containing tetra-tandemer inserts were confirmed by Sanger sequencing (Robart’s Sequencing Facility, London, Ontario, Canada) and their plasmids electroporated into BL21(DE3) *E. coli* cells (Thermofisher) for expression.

Protein production was conducted as previously described, with minor modifications (Vance et al., 2014). Briefly, 1 L of LB broth was inoculated with an overnight 25-mL culture in the presence of 0.1 mg/mL kanamycin, followed by incubation at 37 °C with shaking. At an OD_600_ of 0.6, the 1-L culture was transferred to 23 °C and allowed to reach an OD_600_ of 0.9 before induction with 1.0 mM isopropyl β-D-thiogalactose (IPTG). Following overnight expression, cells from the culture were pelleted and resuspended in 50 mM Tris-HCl (pH 9), 500 mM NaCl, and 2 mM CaCl2. The cells were lysed by sonication, and the cell debris pelleted by centrifugation.

Purification of the tetra-tandemers required a two-step process. 1) Nickel-affinity chromatography: the proteins were incubated with nickel NTA agarose resin (Qiagen) and washed with resuspension buffer + 5 mM imidazole. After 3 column volumes of washing, the protein was eluted using resuspension buffer + 400 mM imidazole. 2) Size-exclusion chromatography: fractions from the nickel-affinity chromatography were pooled and concentrated to 5 mL for injection onto a Supderdex-200 16/60 column (GE Healthcare). Running buffer contained 50 mM Tris-HCl (pH 9), 200 mM NaCl, and 2 mM CaCl_2_. Purity of fractions containing tetra-tandemer was tested using 10% SDS-PAGE.

### 2.2 Calcium titration via circular dichroism spectroscopy

Calcium ions were removed from the protein through dialysis in 10 mM Tris-HCl (pH 9.0), 50 mM NaCl, and 5 mM EDTA. After several changes in this high EDTA buffer, the protein was dialyzed in a low-EDTA version of the same buffer (0.01 mM EDTA) and diluted to 15 µM in this buffer for circular dichroism spectroscopy. Small volumes (1 µL) of concentrated CaCl_2_ (92.5 mM) were added directly to the 200-µL sample in the cuvette to give incremental increases of 0.5 mM Ca^2+^. Twelve scans were taken at 23 °C using a Chirascan CD Spectrometer (Applied Photophysics) for each addition. The scans were averaged, buffer reference-subtracted, and subjected to three-point smoothing using PROVIEWER software. Deconvolution was performed with OLIS SpectralWorks (On-Line Instruments).

### 2.3 Crystallization and structure determination

For crystallography trials, the tetra-tandemers were buffer exchanged into 20 mM Tris-HCl (pH 9) and 10 mM CaCl_2_ and concentrated to 15 mg/mL (*Mh*Lap tetra-tandemer) or 18 mg/mL (*Ah*Lap tetra-tandemer). Microbatch methods were used in which 1 µL of protein was added to 1 µL of different precipitant solutions and covered with 11 µL of paraffin oil. Following optimization from initial crystal hits, both constructs formed well-diffracting, needle-like crystals, but under different conditions. *Mh*Lap tetra-tandemer crystallized in 0.1 M sodium acetate (pH 5.0), 0.5 M sodium chloride, and 20% PEG 6000; *Ah*Lap tetra-tandemer crystallized in 0.1 M sodium acetate (pH 4.6), and 9 – 13% PEG 20, 000.

Structures for both *Mh*Lap and *Ah*Lap tetra-tandemers were solved using calcium phasing. For the *Mh*Lap tetra-tandemer, the single-wavelength anomalous diffraction datasets were collected at a wavelength of 1.77121 Å on CLSID08-1 beamline at the Canadian Light Source (CLS). For the *Ah*Lap tetra-tandemer, a single-wavelength anomalous diffraction dataset was collected on a home X-ray source diffractometer equipped with a chromium rotating anode producing X-rays at a wavelength of 2.2909 Å; a subsequent high-resolution native dataset was collected on CLSID08-1 beamline at the CLS. The datasets were indexed and integrated using XDS (Kabsch, 2010) and scaled and merged by AIMLESS in CCP4 suite (Evans, 2006, Winn et al., 2011). The Ca^2+^-SAD datasets for *Mh*Lap and *Ah*Lap tetra-tandemers were run through Phenix-AutoSol (Liebschner et al., 2019, Terwilliger et al., 2009). The initial phases were calculated at 2.5 Å resolution for both proteins, and then extended to 2.0 Å for the *Mh*Lap tetra-tandemer, followed by Phenix-Autobuild (Terwilliger et al., 2008), BUCCANEER autobuild in CCP4 suite (Cowtan, 2006) and manual model-building using COOT (Emsley et al., 2010). At this point, the solved low-resolution model of the *Ah*Lap tetra-tandemer was used as a search model for a molecular replacement solution of the high-resolution data, using Phenix Phaser (McCoy et al., 2007). Both high-resolution structures were refined with Phenix-Refine (Afonine et al., 2012) and Refmac5 (Vagin et al., 2004, Winn et al., 2011). Crystallographic data collection and refinement statistics are summarized in Table 1. Tetra-tandemer structures were submitted to the Protein Data Bank with PDB codes 6XI3 (*Mh*Lap) and 6XI1 (*Ah*Lap).

**Table 1:** Structural parameters for *Mh*Lap and *Ah*Lap tetra-tandemers.

Protein sample	MhLap	AhLap	AhLap
	Calcium derivative	Native	Calcium derivative
*Data collection statistics*			
Diffraction Sources	Synchrotron CLS08ID-1	Synchrotron CLS08ID-1	Home
Wavelength (Å)	1.77123	0.97949	2.29097
No. of molecules in asymmetric unit	one	two	two
Space group	*C*121	*P*1	*P*1
a, b, c (Å)	202.8, 45.6, 58.6	47.7, 50.4, 124.4	48.0, 50.4, 123.2
α, β, γ (°)	90.0, 100.0, 90.0	97.6, 98.2, 101.8	96.9, 98.2, 102.1
Resolution range (Å)*	46.6 – 2.0 (2.05 – 2.0)	48.64 – 1.75 (1.78–1.75)	48.65 – 2.5 (2.61 – 2.51)
*R*_merge_*	0.10 (0.47)	0.08 (0.83)	0.07 (0.17)
*R*_meas_ *	0.11 (0.49)	0.09 (0.96)	0.08 (0.20)
CC _1/2_*	0.999 (0.990)	0.998 (0.859)	0.997 (0.985)
< *I/σ* > (*I*) >*	19.6 (4.2)	10 (1.9)	16.9 (6.7)
Completeness (%)*	99.0 (87.6)	97.3 (95.3)	91.9 (87.9)
Redundancy*	14.1 (11.4)	3.9 (4.0)	3.9 (3.9)
*Refinement statistics*			
Resolution range (Å)	46.6 – 2.0	48.64 – 1.75	
No. of reflections	34,009	103,273	
*R_work_/R_free_*	018/0.23	0.18/0.21	
No. of atoms			
Protein	2638	6050	
CA ion	4	26	
Water	498	974	
Overall B-factors from Wilson plot (Å^2^)	28.2	17.9	
Average B-factor (Å^2^)			
Protein	29.6	13.7	
Water	43.6	35.3	
CA ion	27.9	24.4	
R.m.s. deviations			
Bond length (Å)	0.011	0.011	
Bond angle (°)	1.726	1.574	
Ramachandran plot statistics (%)			
Most favoured region	96.6	97.28	
Additionally allowed regions	3.11	2.72	
PDB ID	6XI3	6XI1	

* The numbers in parentheses are for the highest resolution shell.

### 2.4 Molecular dynamics

Molecular dynamics simulations of *Mh*Lap and *Ah*Lap di-tandemers were undertaken as previously described (Hakim et al., 2013). Briefly, GROMACS was used to place each di-tandemer in a box of virtual waters and perform energy minimization. This was followed by constant-volume and constant-pressure position-restrained molecular dynamics runs, each 0.1 ns long. Unrestrained molecular dynamics runs were then performed, lasting 20 ns. The duration of this simulation is likely not long enough to see the unfolding of protein domains but is long enough to observe relative changes in domain orientation (Mayor et al., 2000). All simulations were run with a fixed temperature of 298 K. The same protocol was followed for di-tandemers where the calcium ions had been removed.

The output topology and trajectory files were loaded into VMD to analyze the positions of the beta sandwiches relative to each other, and how this changed over time. To measure potential bending of the linker between the domains (Peters et al., 2017), an oblique triangle was drawn between three atoms (***Mh*Lap** = Leu_240_Cβ – Val_247_Cβ – Ile_158_Cβ, ***Ah*Lap** = Ile_228_Cβ – Val_264_Cβ – Tyr_300_Cβ), and the obtuse angle was measured at each frame. To measure potential twisting of the linker between the domains, three lines were drawn between four atoms (***Mh*Lap** = Val_294_Cα – Leu_240_Cβ – Ile_158_Cβ – Ser_130_Cα, ***Ah*Lap** = Ile_334_Cα – Tyr_300_Cβ – Ile_228_Cβ – Glu_152_Cα), and the dihedral angle of the system was measured at each frame.

## 3 Results

### 3.1 Sequence analysis of RTX adhesin extender regions

**Fig. 1:**
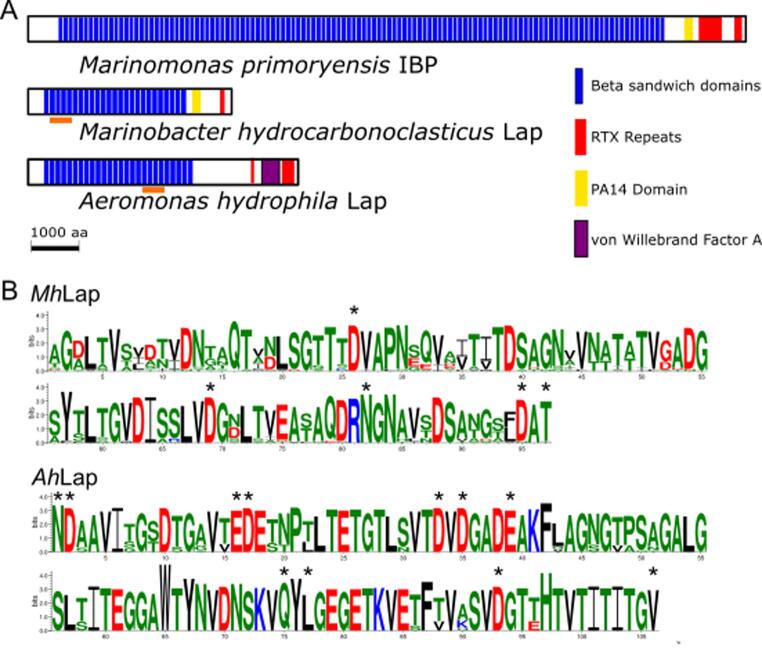
**RTX adhesin domain architecture.** (A) The domain architectures for three exemplar RTX adhesins are shown, with their N termini on the left. Beta sandwiches and RTX repeats – domains that appear in all known RTX adhesins – are coloured blue and red, respectively. Two known domains used for bacterial adhesion are PA14 and vWFA, coloured yellow and purple, respectively. The repeats used for the tetra-tandemer constructs in this study are underlined in orange. (B) Weblogos for all the beta-sandwich repeats present in *Mh*Lap or *Ah*Lap. The residues are colour-coded red for negatively charged, blue for positively charged, black for hydrophobic, and green for polar uncharged/small aliphatic. Asterisks are above calcium-binding residues.

One of the obvious differences in the architectures of the full-length adhesins *Ah*Lap, *Mh*Lap, and the previously characterized *Mp*IBP, is in the number of repeats present in the extender region (Fig. 1A). While the *Mp*IBP has a predicted 120 repeats (which makes up 90% of its 1.5-MDa molecular weight) (Guo et al., 2012), the *Mh*Lap and *Ah*Lap are much shorter, with only ~ 20 repeats each. RTX adhesins with repeat numbers between 20 and 120 are plentiful in the NCBI database, including examples such as LapA (~40), FrhA (~10), SiiE (~50), and RtxA (5 – 40, depending on the strain analysed). It should be noted that adhesin repeat numbers are often underrepresented in genome databases. This occurs when sequencing reads are unable to stretch across large areas of highly repetitive sequence, leading to genome assemblies that fail to show the proper number of repeats. Several such cases have been reported (D’Auria et al., 2008, Guo et al., 2017, Wrobel et al., 2018), and likely many more have yet to be caught. For instance, only a single repeat is reported in the *Ah*Lap sequence from the NJ-35 strain (Dong et al., 2020), which could be an underestimate.

Zeroing in on the sequence of each repeat, the lengths of the beta sandwiches average around 100 amino acids, but the sequence identity between repeats varies wildly. Beta sandwiches within *Mp*IBP share 100% sequence identity even at the DNA level, but the *Mh*Lap and *Ah*Lap repeats show greater variability, with repeat identity ranging between 65 and 90 % and 85 to 95% at the amino-acid level, respectively. A web logo plot of the *Mh*Lap and *Ah*Lap beta sandwiches shows this variability, with *Mh*Lap repeats clearly varying more than those of *Ah*Lap (Fig. 1B). In both cases, a high number of negatively charged aspartate and glutamate residues, as well as their amide derivates, asparagine and glutamine, are present and highly conserved throughout the repeats. Few positively charged residues are present in either protein, although the lysines and arginines that are present are highly conserved.

Taking these sequence observations into account, tetra-tandemer constructs were chosen to maximize the presence of conserved residues, while also providing a spread in sequence identity comparable to the adhesin as a whole (Table 2). Fig. 1A shows from where the tetra-tandemer sequences were drawn within the full-length adhesins, namely towards the N-terminal end for *Mh*Lap and the in latter third of the repeats for *Ah*Lap. Genes encoding these segments were synthesized, using codon redundancy to keep the amino-acid sequence the same as the native protein (Fig. S1), while reducing the sequence identity of the repeats at the DNA level to below 75%, thereby reducing the chance of DNA recombination in *E. coli* (Bzymek and Lovett, 2001).

**Table 2:** Sequence identity of the beta-sandwich repeats within and between RTX adhesins.

	*Mp*IBP	*Mh*Lap	*Ah*Lap
*Mp*IBP	**100%**	28–36%	19–22%
*Mh*Lap	28–36%	**77**–**87%**	15–23%
*Ah*Lap	19–22%	15–23%	**94**–**98%**

### 3.2 Tetra-tandemers show calcium-related structure changes of varying magnitudes

**Fig. 2:**
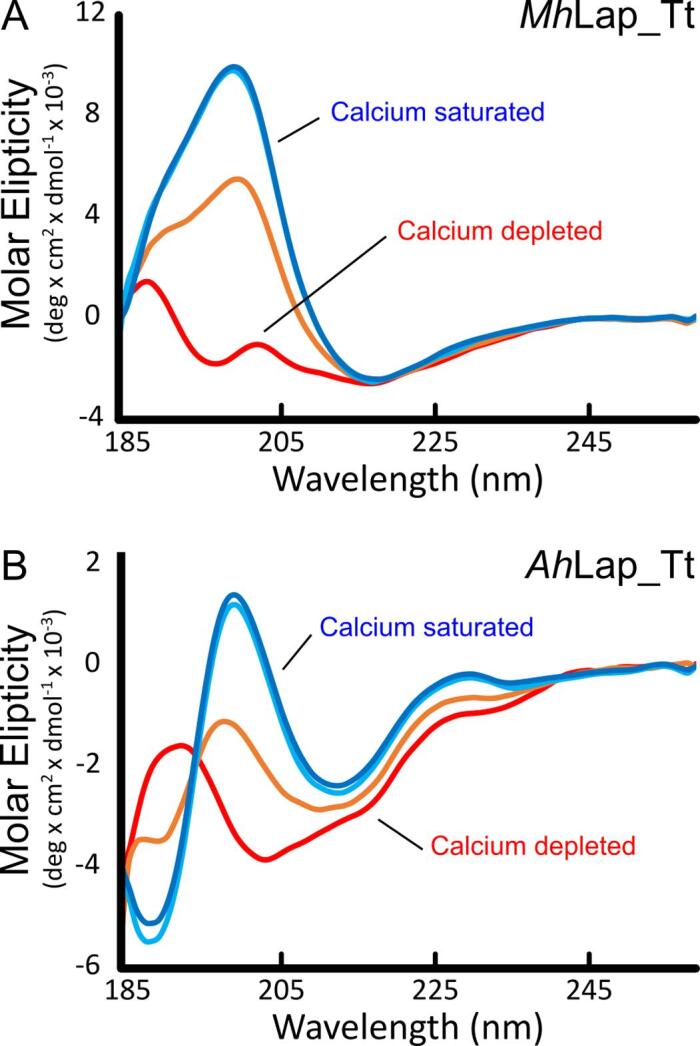
**Circular dichroism spectroscopy.** The CD spectra of a calcium titration for the *Mh*Lap (A) and the *Ah*Lap (B) tetra-tandemers. The titrations began in 0.01 mM EDTA (red), with subsequent additions of CaCl_2_ injected to produce ambient concentrations of 0.5 mM Ca^2+^ (orange), 1 mM Ca^2+^ (cyan) and 2 mM Ca^2+^ (dark blue).

Both the *Mh*Lap and *Ah*Lap tetra-tandemers were expressed in *E. coli* and purified through a combination of nickel-affinity and size-exclusion chromatography. From lysis onwards, both proteins were kept in 2 mM CaCl_2_, in case these tetra-tandemers – like *Mp*IBP – require calcium for proper folding. To test this possibility, the pure proteins were dialyzed against EDTA to remove ambient and bound calcium, and then dialyzed back into a calcium-deprived version of its initial buffer. A calcium titration was then undertaken, with secondary structure changes being monitored through circular dichroism (CD) spectroscopy (Fig. 2).

In the absence of calcium, the *Mh*Lap tetra-tandemer spectrum (red line) shows relatively small changes in molar ellipticity from the baseline, with two minor peaks at 189 and 202 nm, and a minimum at 218 nm (Fig. 2A). Such a spectrum is not easily interpreted as being dominated by a particular secondary structure, instead it suggests there is a roughly even mixture of random coil and beta strand. Addition of 0.5 mM CaCl_2_ led to a drastic change in the spectrum, with a single large maximum appearing at 200 nm (orange line), while its initial minimum at 215 nm is maintained. This change was enhanced by doubling the CaCl_2_ concentration to 1 mM (cyan line), but did not change with further additions of CaCl_2_, indicating a saturation in Ca^2+^-binding. Taken together, these spectra demonstrate that the *Mh*Lap tetra-tandemer develops into a beta-strand-dominated structure upon Ca^2+^ addition, although some structure is present in the absence of calcium.

The *Ah*Lap tetra-tandemer revealed a similar transition from less to more beta structure upon calcium addition, once again saturating at 1 mM CaCl_2_ (Fig. 2B). However, the shape of the *Ah*Lap spectra, as compared to those of *Mh*Lap, was rather different, with the spectrum of the fully-folded *Ah*Lap tetra-tandemer (cyan and dark blue lines) featuring two peaks (major at 200 nm, minor at 230 nm) and two troughs (major at 189 nm, minor at 213 nm), all at much lower molar ellipticity values. Deconvolution of the spectra indicated a lower percentage of beta strand for *Ah*Lap (37%) than for *Mh*Lap (49%) (Table 3), suggesting that the two take on somewhat different folds once calcium-bound, at least at the secondary structure level.

**Table 3:** Deconvolution from CD spectra of *Mh*Lap and *Ah*Lap tetra-tandemers.

	*Mh*Lap			*Ah*Lap		
	0 mM Ca^2+^	1 mM Ca^2+^	Structure	0 mM Ca^2+^	1 mM Ca^2+^	Structure
Alpha helix	3%	3%	0%	0%	0%	0%
310 helix	0%	0%	0%	4%	2%	5%
Beta strand	37%	49%	66%	34%	37%	55%
Turns	18%	22%	26%	17%	21%	28%
Unordered	41%	26%	8%	45%	39%	12%

### 3.3 Tetra-tandemers share similar macro-structure, while the specifics of the folds differ

**Fig. 3:**
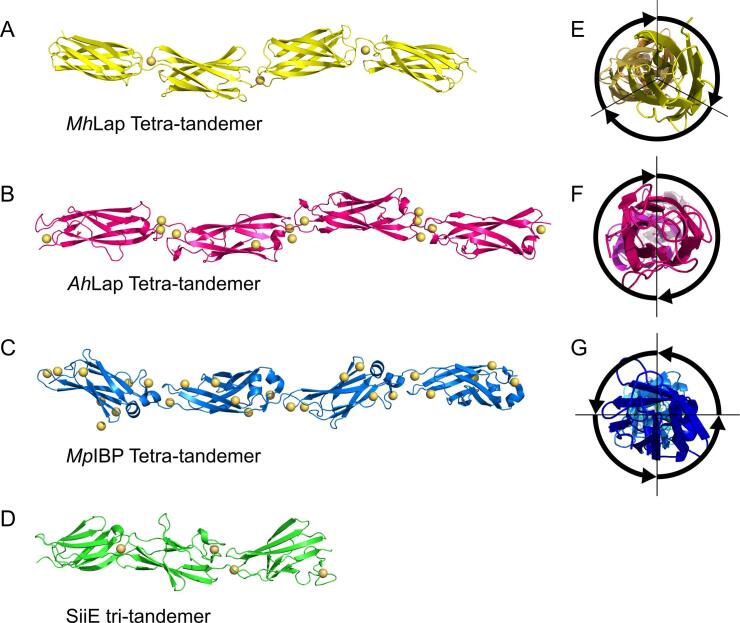
**Tetra-tandemer structures.** Crystal structures of the (A) *Mh*Lap, PDB code: 6XI3 (this study), (B) *Ah*Lap, PDB code: 6XI1 (this study), and (C) *Mp*IBP, PDB code: 4P99 (Vance et al., 2014) tetra-tandemers, as well as the SiiE tri-tandemer, PDB code: 2YN3 (Griessl et al., 2013). Calcium ions are shown as gold spheres. The repeats rotate down a central axis of varying periodicity and direction. Views down that axis from N to C terminus are shown for *Mh*Lap (D), *Ah*Lap (E), and *Mp*IBP (F), along with their direction and the relative position of repeats.

To interrogate the apparent structural difference between the tetra-tandemers, both were crystallized under similar conditions, yielding crystals with asymmetrically elongated unit cells (Table 1). Calcium SAD phasing (Guo et al., 2019a, Guo et al., 2019b) was used to solve the tetra-tandemer structures to 2.0 Å (*Mh*Lap) and 1.75 Å resolution (*Ah*Lap). These structures confirm that the *Mh*Lap (Fig. 3A) and *Ah*Lap (Fig. 3B) tetra-tandemers are made up of four tandem beta-sandwich-like domains that are strung together by short linkers into a linear, rod-like arrangement within the crystal, reminiscent of the previously-solved *Mp*IBP tetra-tandemer (Fig. 3C) and, to a lesser extent, the SiiE tri-tandemer (Fig. 3D). The repeats are rotated relative to each other around the long central axis, producing a handedness to the tetra-tandemers reminiscent of helices. Both the *Mh*Lap (Fig. 3E) and *Ah*Lap (Fig. 3F) structures show right-handed rotations, with pitches of approximately i + 3 and i + 2, respectively. As previously shown, the *Mp*IBP tetra-tandemer rotation stands in contrast as left-handed (Fig. 3G) and can complete a full rotation in five repeats (i + 4). The handedness of SiiE is harder to define, as there are only three domains, the first being distinct in shape and sequence from the others (Griessl et al., 2013).

**Fig. 4:**
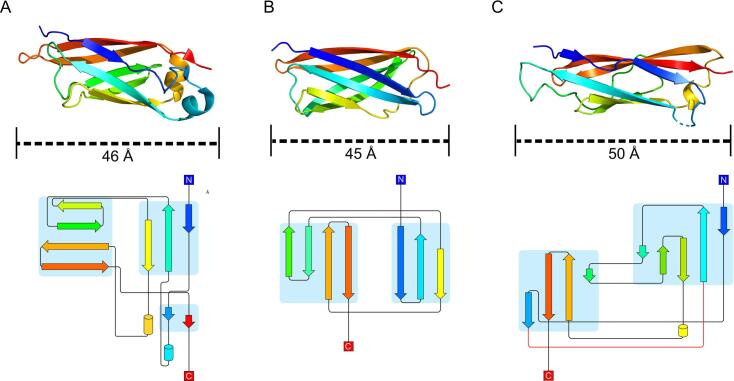
**Structural comparison of beta-sandwich monomers.** Crystal structure and domain topology diagrams for *Mp*IBP (A), *Mh*Lap (B), and *Ah*Lap (C). Structures are coloured by primary sequence to progress from N terminus (blue) to C terminus (red); the strands (arrows) and helices (cylinders) in the topology diagrams (below) are similarly coloured, with the connecting loops in black (or red if not observed in the crystal structure). The length of each monomer is denoted by the black dashed lines below the structures.

Looking closer at the individual repeats for each tetra-tandemer, more differences between the beta sandwiches become apparent. Using the *Mp*IBP as a reference, the 46-Å long repeats are mainly comprised of two antiparallel beta sheets – one three-stranded, the other four-stranded – along with a short, two-stranded, parallel sheet and two alpha helices at the C-terminal end (red) of the domain (Fig. 4A). The 45 Å-long *Mh*Lap repeats are similar yet simpler (Fig. 4B), keeping the two antiparallel beta sheets but lacking the ancillary structural elements present in *Mp*IBP (i.e. the short sheet, and the helices). Despite these differences, the connectivity and partnering of the strands is conserved. Alternatively, the *Ah*Lap repeats possess a more involved connectivity, resulting in 50 Å-long beta sandwiches that are held together by one four-stranded and another five-stranded beta sheet (Fig. 4C). Interestingly, the second strand and the final strand hydrogen bond in a parallel fashion, similar to the short sheet at the C terminus of the *Mp*IBP repeats, while the rest of the strands are antiparallel. As another point of comparison, the beta sandwiches from all three tetra-tandemers are longer than the three solved SiiE beta sandwiches, which range from 30 to 35 Å (Fig. S2). The SiiE extender region is known to possess two distinct beta sandwiches, typified here by Ig50 and Ig51. The two beta sandwiches show remarkably different connectivities, with Ig50 being more similar to the simpler *Mh*Lap (Fig. S2).

Even with the differences in domain folding and relative rotation, the tetra-tandemers (and SiiE) all maintain a similarly elongated conformation. This conservation of macro-structure is in spite of the seemingly flexible interstitial loops between domains, which lack defined secondary structure. All four structures reveal coordinated Ca^2+^ within these regions (Fig. 3), varying in number and coordination pattern, yet apparently conserved in purpose: to rigidify the linkers and project an elongated conformation.

### 3.4 *Mh*Lap coordinates a single Ca^2+^ within the flexible linker regions

Regarding Ca^2+^ coordination, the *Mh*Lap tetra-tandemer is once again the simplest of the three. While the *Mp*IBP tetra-tandemer was found to have many Ca^2+^ coordinated throughout its structure, *Mh*Lap consigns its calcium ions to the linker regions, with only one ion per domain interface. This limited calcium complement may explain the differences seen in CD spectra during calcium titrations, as *Mp*IBP tetra-tandemer loses all beta and alpha characteristics in EDTA (Table S1) (Guo et al., 2013), while *Mh*Lap retains some level of structure (Fig. 2A).

**Fig. 5:**
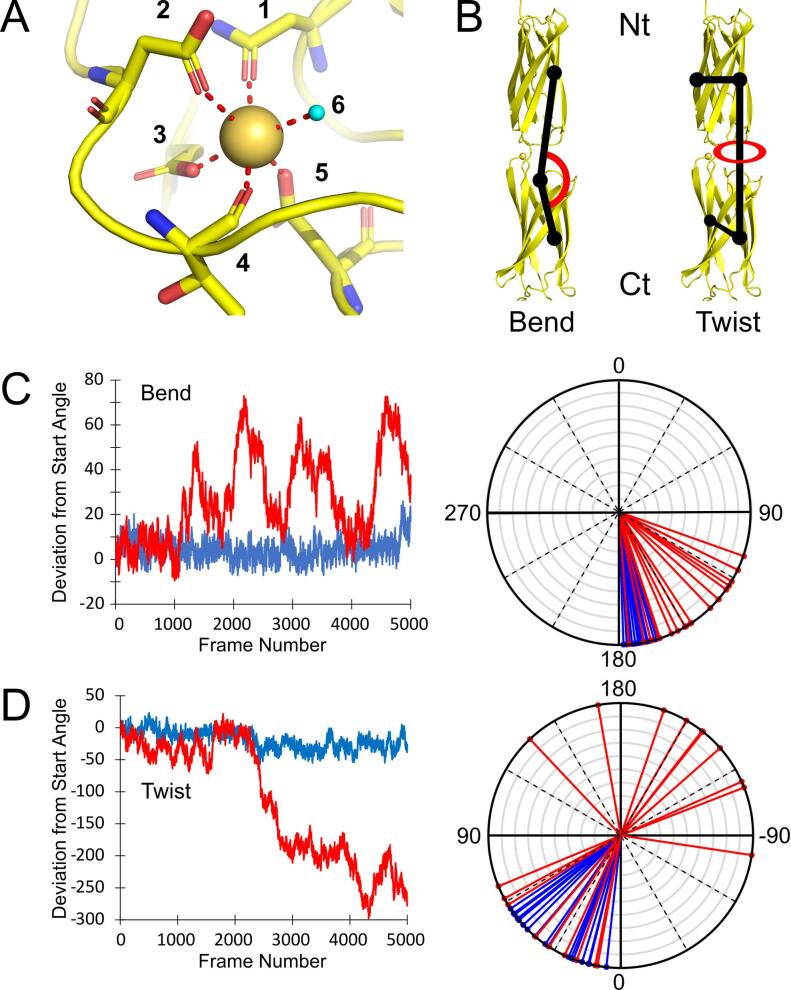
***Mh*Lap calcium coordination and molecular dynamics.** (A) The coordination of inter-repeat Ca^2+^ in the *Mh*Lap tetra-tandemer. Ca^2+^ is shown as a gold sphere, and residues binding to it are numbered as follows: 1 = Asn_205_, 2 = Asp_121_, 3 = Asp_95_, 4 = Thr_123_, 5 = Asp_149_, 6 = water. Water molecules participating in the coordination sphere are shown as cyan spheres. (B) The di-tandemer *in silico* construct used for molecular dynamics simulations. The Bend and Twist angles to be monitored throughout the simulations are shown, and the termini are labelled Nt and Ct. (C) The Bend angle throughout the simulations, depicted in two ways. Left: a plot of deviation from start angle over frame number. Right: a radial plot where the Bend angle every 250 frames is denoted by a line. Simulations with (blue) and without (red) the coordinated Ca^2+^ ions are shown. (D) The Twist angle throughout the simulations, depicted the same way as the Bend angle.

Each calcium in the *Mh*Lap tetra-tandemer structure is bound in a similar manner (Fig. 5), forming an octahedral coordination sphere comprised of five protein contacts and one water molecule. The protein contacts are made by the sidechains of one asparagine and three aspartate residues, as well as a single backbone carbonyl (Fig. 5A). These Asn/Asp residues are conserved throughout the entirety of the full-length *Mh*Lap extender region (Fig. 1B), though there are many other like residues that are equally-well conserved that do not appear to be involved in calcium coordination.

To analyze the potential rigidifying properties of these interstitial calcium ions, molecular dynamics simulations on a di-tandemer of the *Mh*Lap were undertaken with and without the Ca^2+^ present. Two angles were measured throughout the 5000 frames (Fig. 5B), one to assay bending of the domains at the linker region (Bend), the other to assay rotation of the repeats about the central axis (Twist). In the presence of calcium, both angles underwent minor changes over the course of the simulation (blue tracing in Fig. 5C and D). The di-tandemer maintained a rod-like structure, with the Bend angle between the two domains never deviating over 30° relative to the start frame; the two domains saw slightly more twisting over the simulation, maintaining a ~ 60° wedge.

**Fig. 6:**
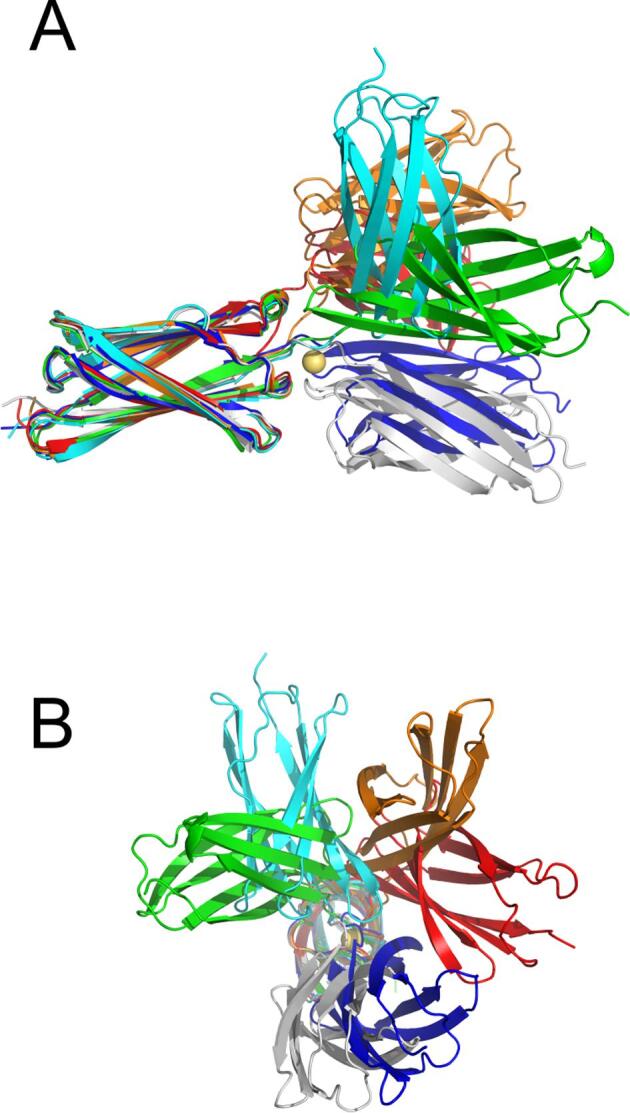
**Frames from the *Mh*Lap No-Ca^2+^ molecular dynamics simulation.** The first repeat of the di-tandemer construct at frames 1000 (blue), 2200 (cyan), 3000 (green), 4000 (orange), and 4500 (red) were aligned against the first repeat of the truncated crystal structure (white). Both a side view (A) and a view down the central axis (B) are shown.

Removing the calcium introduced major variability in both angles. With regards to bending, the two domains oscillated between an angle close to that in the start frame and a second angle ~ 70° from the start frame (red tracing in Fig. 5C, left), bending back and forth over the course of the 20 ns simulation four times. At points, the two domains approach a perpendicular orientation relative to each other (Fig. 5C, right). The twist dihedral angle drastically changes from the start frame to approach a ~ 300° deviation (Fig. 5D, left). Indeed, the two domains can sample almost the entire 360° rotation throughout the simulation (Fig. 5D, right). Visualizing the protein at select frames makes the magnitude of these structural changes strikingly clear (Fig. 6). The di-tandemer at frame 1000 (blue) is very similar to the crystal structure’s orientation (light gray). But as the simulation progresses, the two beta sandwiches sample a wide variety of relative angles (cyan through red). The aforementioned perpendicular orientation can be seen at frame 2200 (cyan).

### 3.5 *Ah*Lap uses three Ca^2+^ to maintain its rod-like conformation

**Fig. 7:**
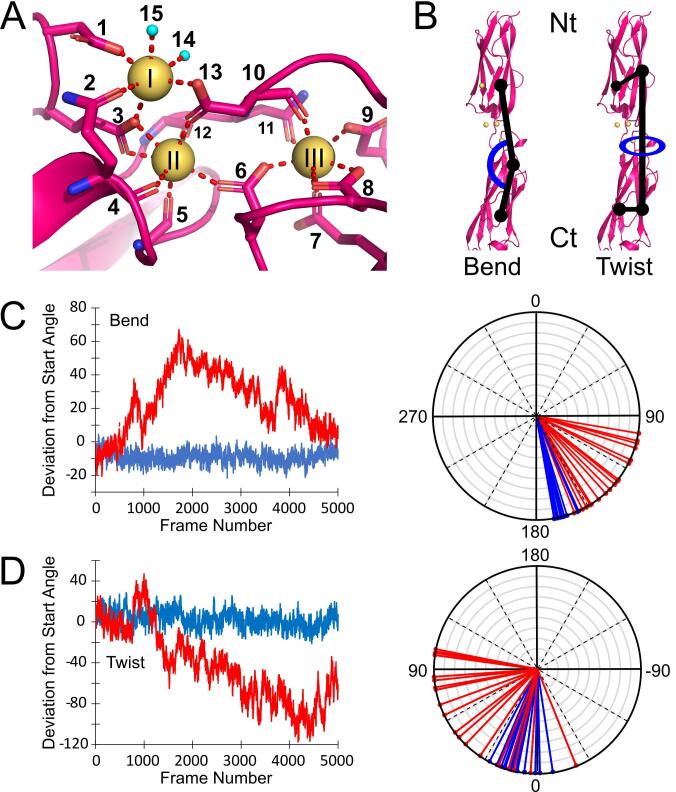
***Ah*Lap calcium coordination and molecular dynamics.** (A) The coordination of inter-repeat Ca^2+^ in the *Ah*Lap tetra-tandemer. Ca^2+^ are shown as gold spheres, and residues binding to them are numbered as follows: 1 = Asp_144_, 2 = Gln_202_, 3 = Glu_143_, 4 = Gln_202_, 5 = Leu_204_, 6 = Asp_268_, 7 = Glu_272_, 8 = Asp_266_, 9 = Asp_326_, 10 = Asp_235_, 11 = Asn_234_, 12 = Val_233_, 13 = Asp_235_, 14 = water, 15 = water. Water molecules participating in the coordination sphere are shown as cyan spheres. (B) The di-tandemer *in silico* construct used for molecular dynamics simulations. The Bend and Twist angles to be monitored throughout the simulations are shown, and the termini are labelled as in Fig. 5. (C) The Bend angle throughout the simulations, depicted in two ways. Left: a plot of deviation from start angle over frame number. Right: a radial plot where the Bend angle every 250 frames is denoted by a line. Simulations with (blue) and without (red) the coordinated Ca^2+^ ions are shown. (D) The Twist angle throughout the simulations, depicted the same way as the Bend angle.

The *Ah*Lap tetra-tandemer coordinates many more calcium ions throughout its length than does *Mh*Lap, although the number of ions per repeat is inconsistent. Each inter-domain interface contains, at minimum, three highly coordinated Ca^2+^ (Fig. 7A), labelled as calciums I, II, and III. Of the three, calcium I has the fewest coordinate bonds to protein-based ligands, with two positions in its six-ligand octahedral coordination sphere being taken up by water molecules. Both calciums II and III have pentagonal bipyramid coordination spheres, comprised of seven protein-based ligands each. The three Ca^2+^ are all coordinated by a combination of Asp, Asn, and Glu sidechains that are conserved in every repeat of the full-length adhesin (except the Glu in position 7, which is a comparable Gln for five repeats), as well as backbone carbonyl groups (Table 4). Interestingly, several acidic residues (namely ligands 3, 6, and 13 in Fig. 7A) use their bidentate sidechains to coordinate two separate calcium ions at the same time, making calciums I, II and III part of an interconnected coordination network.

**Table 4:** Coordinating ligands of inter-domain Ca^2+^ ions.

*Mh*Lap Tetra-tandemer			*Ah*Lap Tetra-tandemer		
	Residue	Number		Residue	Number
1	Asn _sidechain_	205/302/399	1	Asp _sidechain_	38/144/250
2	Asp _sidechain_	121/218/315	2	Gln _sidechain_	96/202/308
3	Asp _sidechain_	95/192/289	3	Glu _sidechain_	37/143/249
4	Thr _backbone_	123/220/317	4	Gln _backbone_	96/202/308
5	Asp _sidechain_	149/246/343	5	Leu _backbone_	98/204/310
6	Water		6	Asp _sidechain_	162/268/374
			7	Glu _sidechain_	166/272/378
			8	Asp _sidechain_	160/266/372
			9	Asp _sidechain_	220/326/432
			10	Asp _backbone_	129/235/341
			11	Asn _sidechain_	128/234/340
			12	Val_backbone_	127/233/339
			13	Asp _sidechain_	129/235/341
			14	Water	
			15	Water	

Besides these three consistent calcium ions, additional Ca^2+^ are present in select repeats. The *Ah*Lap structure was solved with two tetra-tandemers in the asymmetric unit, meaning that the structure holds eight repeat domains with six replicate interfaces between them. Of those six, four of them hold an additional calcium (Fig. S3). These calcium ions are held in place by only two protein-based ligands, with the rest of the octahedral coordination sphere made up of water ligands. This minimal connection to the protein explains the inconsistent presence of the ion in the structure. A single additional calcium is found outside of the domain interfaces, attached to the side of the second repeat in chain A. As expected, this calcium has fewer protein contacts, and is only coordinated by two protein-based ligands (Fig. S4). Whether these additional ions are present *in vivo* is uncertain, though the residues that coordinate them are highly conserved throughout the entirety of *Ah*Lap.

Molecular dynamics simulations were run on an *Ah*Lap di-tandemer in the presence and absence of calcium, as was done for *Mh*Lap. Again, two similar angles were measured throughout to observe the relative twisting and bending of the domains (Fig. 7B). Only the three conserved calcium ions were included in the calcium-containing run, which produced an expected minimal amount of bending or twisting between the domains (blue tracing in Fig. 7C and D). In fact, the *Ah*Lap tetra-tandemer appears more rigid than the *Mh*Lap, with both angles remaining closer to the start angle over the entirety of the run.

**Fig. 8:**
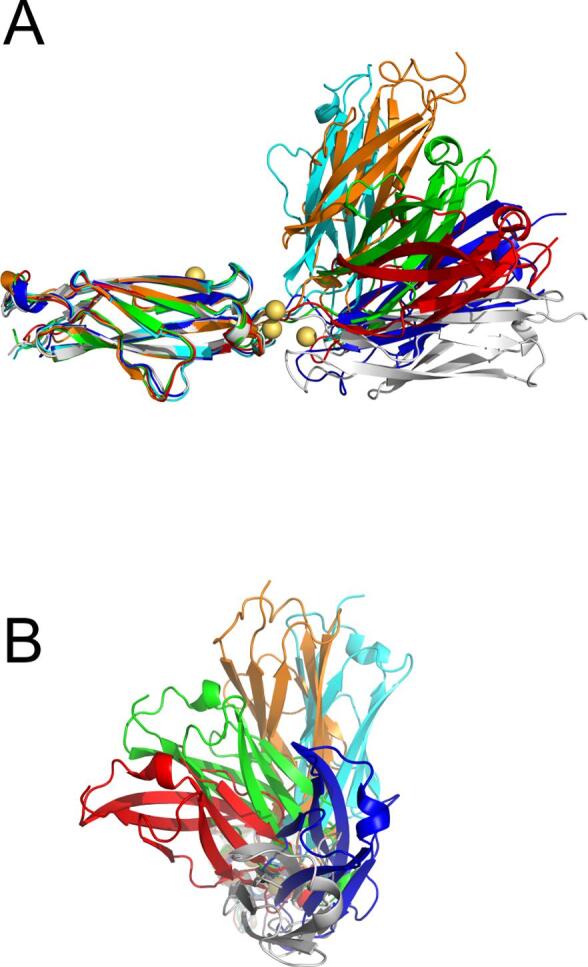
**Frames from the *Ah*Lap No-Ca^2+^ molecular dynamics simulation.** The first repeat of the di-tandemer construct at frames 500 (blue), 2000 (cyan), 3000 (green), 4000 (orange), and 5000 (red) were aligned against the first repeat of the truncated crystal structure (white). Both a side view (A) and a view down the central axis (B) are shown.

Removal of the conserved calcium ions once again led to a stark increase in variability for both angles. The two domains were able to bend at the linker region to reach a 70° deviation from the start angle (red tracing in Fig. 7C, left) almost becoming perpendicular to each other at times (Fig. 7C, right). Interestingly, this change was less frequent than for *Mh*Lap, with a single oscillation taking the whole simulation, while *Mh*Lap underwent several throughout its run. The twisting between the *Ah*Lap domains was not as severe as for *Mh*Lap, deviating by a striking 120° (Fig. 5D), which is far less than *Mh*Lap’s ~ 300° wedge. Aligning select frames once again showcases the variety of orientations taken up by the di-tandemer (Fig. 8), especially in frames 1000 (cyan) and 4000 (orange) where the repeats are almost perpendicular to each other. This comparison also visualizes how the *Ah*Lap di-tandemer is apparently less flexible than *Mh*Lap, limiting the orientations it can explore.

In summary, a similar trend is observed from both simulations: the absence of calcium removes the stable rod-like structure of these tandem beta-sandwich repeats, leading to a much greater freedom in domain orientation.

## 4 Discussion:

### 4.1 Calcium coordination as a prerequisite for RTX-mediated biofilms

Recently, Dong *et al.* published a paper detailing hyper biofilm-forming mutants in *A. hydrophila* strains that infect fish (Dong et al., 2020). It was found that an overproduction of *Ah*Lap (named against convention within the study as RmpA) was responsible for this new phenotype, adding *Ah*Lap to the growing list of RTX adhesins vital to biofilm formation (Guo et al., 2017, Hinsa et al., 2003, Martínez-Gil et al., 2010, Syed et al., 2009). While deletions of *Ah*Lap removed the hyper biofilm-forming phenotype as expected, so too did the removal of Ca^2+^ from the solution via chelating agents (Dong et al., 2020). This result mirrors other such experiments on RTX adhesin-mediated behaviour, like studies on the RTX adhesin SiiE in *Salmonella enterica*, where the removal of calcium greatly reduced invasion of the bacteria into polarized epithelial cells (Peters et al., 2017).

The experiments on the auto-aggregating *Ah*Lap mutants were unable to pinpoint exactly what effect Ca^2+^ removal had on the adhesin-mediated biofilm. Fortunately, previous research into the biochemistry of RTX proteins (including this study) can offer some suggestions. Studies have shown that the relationship between RTX adhesins and Ca^2+^ is a complex and multifaceted one, impacting proper secretion, folding, orientation, and adhesin-substrate contacts. As indicated by CD spectroscopy results both here and elsewhere (Guo et al., 2017, 2013), coordinated calcium ions are key in the folding of most RTX adhesin domains, which impacts everything from the protease-resistance of the adhesins (Peters et al., 2017) to their localization on the cell surface via the type 1 secretion system (T1SS). T1SS-secreted proteins must remain unfolded for transport through the dual membranes of Gram-negative bacteria, and while there are some that require chaperone proteins (Delepelaire and Wandersman, 1998), it is thought that the adhesins rely on calcium-dependent folding to remain intracellularly unfolded (Bumba et al., 2016). Interestingly, our results show that certain domains from RTX adhesins are not completely unfolded in the absence of Ca^2+^, as have previous studies on domains from *Mh*Lap (Vance et al., 2019) and SiiE (Peters et al., 2017). The calcium-free partial structures of such RTX adhesin domains are likely unstable and weakly held together, as was shown in studies on the thermal stability of calcium-free mutants of SiiE beta sandwiches (Peters et al., 2017) and single-molecule force microscopy experiments on calcium-deprived *Mp*IBP octa-tandemers (Oude Vrielink et al., 2017). As such, these intracellular structures are expected to easily pull apart during secretion through the pores of the T1SS, refolding into their strong calcium-bound structures as they reach the extracellular space.

Examination of the adhesion domains from *Mh*Lap (Vance et al., 2019) and *Mp*IBP (Guo et al., 2017) have demonstrated how Ca^2+^ is also at the forefront of adhesin-substrate interactions, where the ions coordinate ligands from both adhesin and glycan/peptide substrates. However, these contacts could not be established if calcium ions were not also involved in determining the proper orientation of the adhesin’s domains relative to each other in 3-D space. While inter-domain calcium ions are likely present throughout the adhesin, perhaps orienting the adhesion domains in the C-terminal regions into particular macrostructures (Guo et al., 2017, Guo et al., 2019a, Guo et al., 2019b), certainly this Ca^2+^-mediated ordering of domains is most obvious in the rigidification of the extender region. Demonstrated clearly by the MD simulations of *Ah*Lap in the absence of calcium, the adhesin’s stalk could not channel the exploration of the adhesion domains to substrates away from the bacterium’s own surface if the domains could bend and twist as freely as they do in the absence of Ca^2+^.

### 4.2 Calcium-induced rigidification: A conserved or convergent strategy?

The importance of a rigid extender region is highlighted by their presence throughout adhesin families, both RTX and otherwise. Filamentous adhesins, such as the type I pili from *E. coli*, connect many Ig-like monomers into long threads that extend the sugar-binding tip towards a substrate (Schilling et al., 2001). The type Ve autotransporter adhesins from Gram-negative bacteria also use beta-sandwich extenders (Tsai et al., 2010), as do the Gram-positive biofilm-associated proteins (Baps) (Cucarella et al., 2001). Yet, while rigidification appears to be a prerequisite for all useful extension regions, the use of calcium-induced rigidification is not universal. For example, neither pili nor autotransporter adhesins require calcium for rigidification. Instead, type I pili assemble their monomers into a stiff helical shape (Hospenthal et al., 2017, Sauer et al., 2004), and invasins use linkers rich in proline residues (Palumbo and Wang, 2006).

Is calcium rigidification conserved throughout all RTX adhesins? Our studies here have increased the number of linked extender region structures in RTX adhesins from two to four. In all four structures, the beta sandwiches coordinate calcium ions in the linker regions between domains. The weight of evidence suggests that these calcium ions are used for rigidification, as seen for SiiE by electron microscopy, small angle x-ray scattering (SAXS), and molecular dynamics simulations (Griessl et al., 2013, Peters et al., 2017); for *Mp*IBP by SAXS and single-molecule force microscopy (Oude Vrielink et al., 2017, Vance et al., 2014), and now for both the *Mh*Lap and the *Ah*Lap by molecular dynamics. Beyond the studied examples, sequences of predicted RTX adhesins within the NCBI database show a consistent excess of negatively charged residues, suggesting that this calcium-rigidification strategy is widely conserved.

However, while the outcome remains the same, the specifics of how calcium stabilizes the rod-like structure of these tandem repeats vary. Between the three tetra-tandemers and the heavily studied SiiE tri-tandemer, both the number of calcium ions present between repeats and the coordinating residues of these ions are remarkably different. Along with the low sequence identity between different adhesin’s beta-sandwich domains, one begins to question whether this calcium-rigidification strategy is really conserved from a progenitor adhesin in all cases, or – as an alternative theory –the strategy arose convergently between different species, finding similar solutions to the need for rigidification and extension but via different origins. The profound differences in the *Ah*Lap monomer (both sequence identity and connectivity) and calcium coordination relative to both *Mp*IBP and *Mh*Lap would seem to support evolutionary convergence.

## 5 Conclusions

Expansive extender regions made up of tandem beta-sandwich repeats are a defining feature of the biofilm-associated RTX adhesins. Here, segments of extender regions – one from an oil-eating bacterium, the other an opportunistic pathogen of fish and mammals – were structurally characterized. In keeping with the known importance of calcium for RTX adhesins, both extender segments were found to coordinate Ca^2+^ in a manner that maintains the otherwise unwieldy string of beta sandwiches as a rigid and rod-like structure. However, there were striking differences in the structure and coordination strategy between these proteins, raising questions as to the evolutionary origin of these regions in supposedly homologous adhesins across the bacterial domain.

Research into this expanding family of adhesins continues to show the importance of calcium, and the specifics of how the ions are integrated into the extracellular protein’s functions are becoming more complicated. As strategies to control RTX adhesin-mediated biofilms mature, the keystone calcium ion will become an obvious target. While no one solution for all biofilms is likely to exist, the dependence of RTX adhesins on calcium as a potent connector may come close.

## 6 Data availability

X-ray crystal structure coordinates solved in this study have been deposited in the Protein Data Bank with accession codes 6XI3 (*Mh*Lap) and 6XI1 (*Ah*Lap). Data that support the findings of this study are available from the corresponding author P.L.D. upon reasonable request.

## CRediT authorship contribution statement

**Tyler D.R. Vance:** Conceptualization, Software, Investigation, Visualization, Supervision. **Qilu Ye:** Validation, Investigation. **Brigid Conroy:** Investigation. **Peter L. Davies:** Conceptualization, Resources, Supervision, Project administration, Funding acquisition.

## Declaration of Competing Interest

The authors declare that they have no known competing financial interests or personal relationships that could have appeared to influence the work reported in this paper.
