# Supplementary material for: Essential role of calcium in extending RTX adhesins to their target
Source: J Struct Biol X. 2020 Sep 8;4:100036. doi: 10.1016/j.yjsbx.2020.100036 (PMC7493085; doi:10.1016/j.yjsbx.2020.100036)
Supplement: Supplementary data 2 [file mmc2.docx]

Appendix A: Supplementary information for

**Essential role of calcium in extending RTX adhesins to their target**

Vance T.D.R. , Ye Q. , Conroy B. , and Davies P.L. ^*^

*** Corresponding author:**

Peter L. Davies, Department of Biomedical and Molecular Sciences, Queen’s University

18 Stuart Street Kingston ON, Canada, K7L 3N6

Email: [daviesp@queensu.ca](mailto:daviesp@queensu.ca)

1-613-533-2983

*Web page: https://www.peterldavies.com/*

**A) Supplementary Figures**

>*Mh*lap_Tt [DNA sequence]

**CAT ATG** AGC TTT GAT GCA ACC GCA GGC GCA CTG ACC GTT AGC CTG GAT ACC GTT GAT AAT ACC GCA CAG ACC GCA AAT CTG AGC GGC ACC ACC ACC GAT GTT GCA CCG AAT GAA CAG GTT GCA ATT ACC ATT ACC GAT AGC GCA GGC AAT ATT GTT AAT GCA ATT GCA ACC GTT GGT GCC GAT GGT AGC TAT AGC CTG ACC GGT GTT GAT ATT AGC AGC CTG GTT GAT GGT AGT CTG ACC GTG GAA GCA AGC GCA CAG GAT CGT AAT GGT AAT GCC CTG ACC GAT AGT GCA AAT GGT GCA CTG GAT GCA ACA GCC GGT GAT CTG ACA GTT AGC GTT GGC ACC ATT GAT AAC ACA GCC CAG ACA GTT AAT CTG TCA GGT ACA ACC ACA GAC GTA GCC CCT AAT GGT CAG GTG GCC ATT ACA ATG ACC GAT TCA GCA GGT AAC ATT GTG AAT GCC ACC GCC ACC GTG GGA GCA GAT GGT TCA TAT TCA CTG ACA GGC GTG GAT ATT TCA AGT CTG GTG GAT GGC GAT CTG ACG GTT GAA GCA TCA GCC CAG GAT CGC AAC GGC AAT GCG GTT AGT GAT AGC GCC AAT GGT ACA TTT GAT GCC ACT GCG GGT GAT TTA ACC GTG AGC GTT GAT ACC GTG GAT TCT ACA GCG CAG ACG GCA AAC CTG TCA GGC ACG ACA ACG GAT GTT GCC CTG AAT AGC CAG GTT GAC CTG ACA GTG ACA GAC AGT GCA GGT AAT GTT GTT ACC GCA ACC ACC ACA GTA GGT GCG GAT GGC AGT TAT TCT TTA ACC GGT GTG GAC ATT TCT TCA CTG GTG GAC GGT AAC TTA ACC GTT GAA GCG ACA GCT CAA GAC CGT AAT GGC AAC GCA GTT TCA GAT AGT GCC GCA GGC AGT CTG GAT GCG ACC ACC GGT GCG CTG ACT GTT TCA CTG GAC ACA GTG GAT AAT GCT GCC CAG ACG GTG GAT CTG AGT GGT ACA ACA GCG GAT GTG GCT CCG AAT TCA CAG GTT AAT GTG ACC ATT ACA GAT TCA ACC GGT AAT GTG GTG AAT GCG ATT ACC ACG GTT GGC GCT GAT GGC AGC TAC TCT CTG ACT GGC GTT GAC ATC AGC TCA CTG GTA GAT GGT GAC TTG ACA GTA GAA GCG TCA GCG CAG GGA CGC AAT GGA AAC GCG CTG ACG GAT TCT GCG AAC GGT GCC CTG GAC GCC ACC **TAA** **CTCGAG**

>*Ah*Lap_Tt [DNA sequence]

**CAT ATG** AAT GAT GCA GCA GTT ATT ACC GGT AGC GAT ACC GGT GCA GTT ACC GAA GAT GAA AGC ACA CCG CTG CTG ACC GAA ACC GGC ACC CTG AGC GTT ACC GAT GTT GAT GGT GCA GAT GAA GCA AAA TTT CAG GCA GGT AAT GGC ACC CCG AGT GCC GGT GCA CTG GGT AGC CTG ACC ATT ACG GAA GGT GGT GCA TGG ACC TAT AAC GTT GAT AAT TCA AAA GTT CAG TAT CTG GGT GAA GGT GAA ACC AAA GTT GAA ACC TTT ACC GTT GCA AGC GTT GAT GGC ACC ACC CAT ACC GTT ACC ATT ACC ATC ACC GGT GTT AAC GAT GCT GCC GTG ATT ACC GGC TCA GAT ACA GGT GCC GTG ACA GAG GAT GAA TCA AAT CCG ACC CTG ACA GAA ACA GGT ACA CTG TCA GTG ACA GAT GTG GAC GGT GCC GAC GAG GCC AAA TTC CTG GCA GGC AAT GGT ACG CCG TCA GCG GGT GCC CTG GGT TCA CTG ACA ATC ACC GAA GGC GGA GCC TGG ACC TAC AAT GTG GAT AAC AGC AAA GTG CAG TAT TTA GGC GAA GGC GAG ACA AAA GTG GAA ACA TTC ACC GTG GCA TCA GTG GAT GGT ACA ACA CAT ACA GTG ACA ATT ACA ATT ACG GGT GTA AAT GAC GCA GCG GTG ATT AGT GGT TCT GAT ACT GGC GCT GTG ACG GAA GAT GAG AGT ACC CCG TTA CTG ACG GAA ACG GGA ACG CTG TCT GTT ACG GAC GTG GAT GGC GCT GAT GAG GCG AAA TTC TTA GCC GGT AAT GGT GTT GCC AGC AAT GGT GCG CTG GGC TCT TTA ACC ATC ACA GAG GGT GGA GCG TGG ACA TAT AAC GTA GAC AAT AGT AAA GTG CAA TAC CTG GGC GAG GGC GAA ACG AAG GTT GAG ACA TTT ACA GTG GCC AGT GTG GAT GGA ACG ACC CAC ACG GTG ACC ATC ACG ATT ACA GGC GTT AAC GAC GCA GCT GTT ATT TCA GGT TCC GAT ACG GGT GCG GTA ACT GAG GAC GAA ACC AAT CCG CTG TTA ACG GAA ACC GGT ACA TTA AGT GTG ACT GAT GTA GAT GGA GCG GAC GAA GCG AAG TTT TTA GCA GGT AAC GGT ACA CCG TCT GCA GGC GCA TTA GGT TCT CTG ACG ATT ACC GAG GGA GGC GCT TGG ACG TAC AAC GTC GAC AAT AGC AAG GTA CAG TAT CTT GGA GAG GGT GAG ACT AAG GTA GAA ACG TTT ACG GTG GCC TCA GTT GAC GGC ACG ACA CAC ACA GTT ACG ATA ACC ATT ACT GGG GTT AAT GAT GGC GCA **TAA CTCGAG**

>*Mh*lap_Tt [amino-acid sequence]

**MASSHHHHHHSSGLVPRGSHMSFDATAGALTVSLDTVDNTAQTANLSGTTTDVAPNEQVAITITDSAGNIVNAIATVGADGSYSLTGVDISSLVDGSLTVEASAQDRNGNALTDSANGALDATAGDLTVSVGTIDNTAQTVNLSGTTTDVAPNGQVAITMTDSAGNIVNATATVGADGSYSLTGVDISSLVDGDLTVEASAQDRNGNAVSDSANGTFDATAGDLTVSVDTVDSTAQTANLSGTTTDVALNSQVDLTVTDSAGNVVTATTTVGADGSYSLTGVDISSLVDGNLTVEATAQDRNGNAVSDSAAGSLDATTGALTVSLDTVDNAAQTVDLSGTTADVAPNSQVNVTITDSTGNVVNAITTVGADGSYSLTGVDISSLVDGDLTVEASAQGRNGNALTDSANGALDAT**

>*Ah*Lap_Tt [amino-acid sequence]

**MASSHHHHHHSSGLVPRGSHMNDAAVITGSDTGAVTEDESTPLLTETGTLSVTDVDGADEAKFQAGNGTPSAGALGSLTITEGGAWTYNVDNSKVQYLGEGETKVETFTVASVDGTTHTVTITITGVNDAAVITGSDTGAVTEDESNPTLTETGTLSVTDVDGADEAKFLAGNGTPSAGALGSLTITEGGAWTYNVDNSKVQYLGEGETKVETFTVASVDGTTHTVTITITGVNDAAVISGSDTGAVTEDESTPLLTETGTLSVTDVDGADEAKFLAGNGVASNGALGSLTITEGGAWTYNVDNSKVQYLGEGETKVETFTVASVDGTTHTVTITITGVNDAAVISGSDTGAVTEDETNPLLTETGTLSVTDVDGADEAKFLAGNGTPSAGALGSLTITEGGAWTYNVDNSKVQYLGEGETKVETFTVASVDGTTHTVTITITGVNDGA**

**Figure S1. The DNA and amino-acid sequences for *Mh*Lap and *Ah*Lap tetra-tandemers.** Repeats oscillate between non-highlighted and highlighted (yellow=*Mh*Lap, purple=*Ah*Lap). The start and stop of the repeats as presented are derived from the solved structures. Sequences derived from the plasmid are coloured gray. Restriction enzyme sites are highlighted (blue = *Nde1*, red = *Xho1*). Stop codons are coloured red.


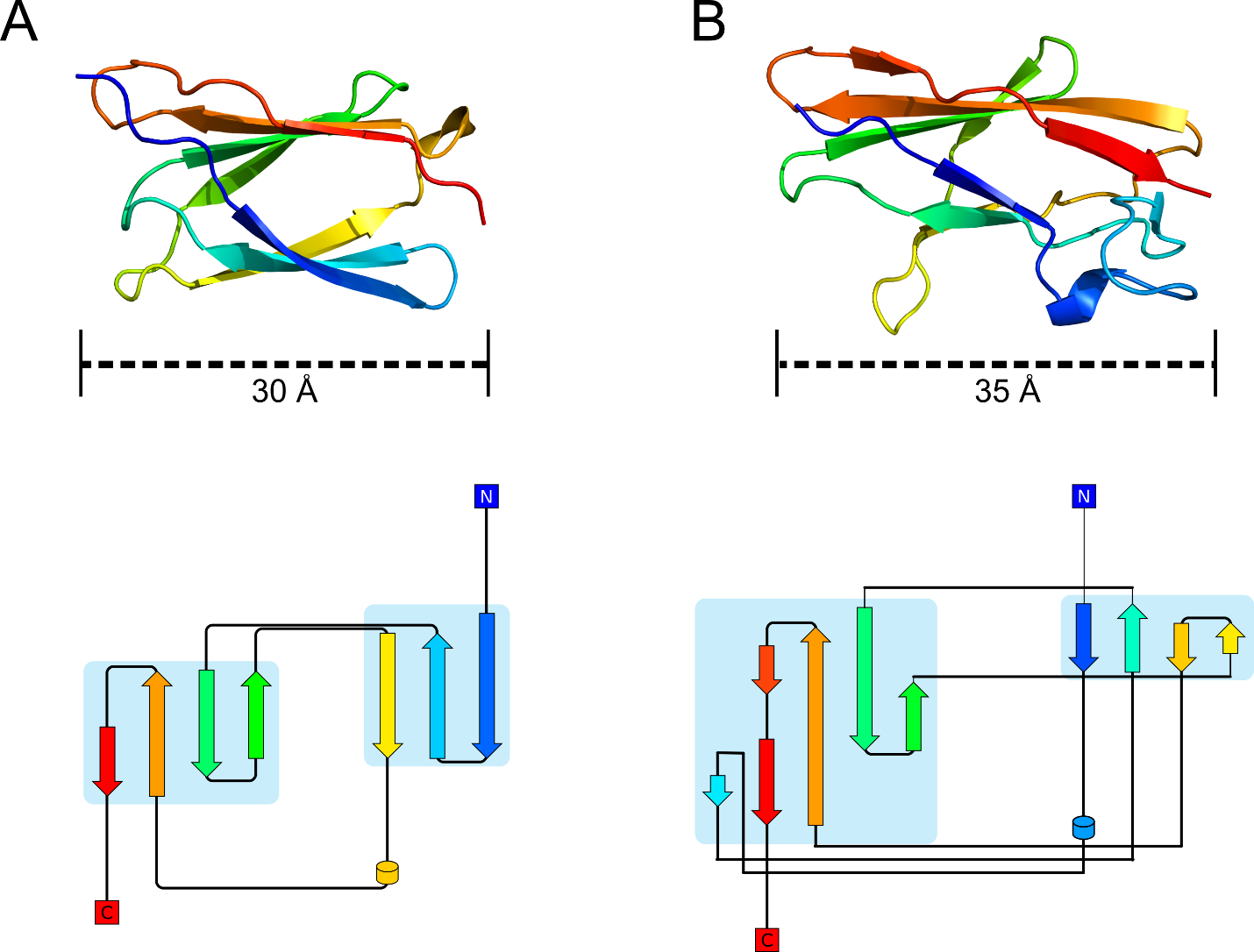


**Figure S2. Structural comparison of SiiE monomers.** Crystal structure and domain topology diagrams for SiiE Ig50 (A), and SiiE Ig51 (B). Structures are coloured by primary sequence to progress from N terminus (blue) to C terminus (red); the strands (arrows) and helices (cylinders) in the topology diagrams (below) are similarly coloured, with the connecting loops in black. The length of each monomer is denoted by the black dashed lines below the structures.


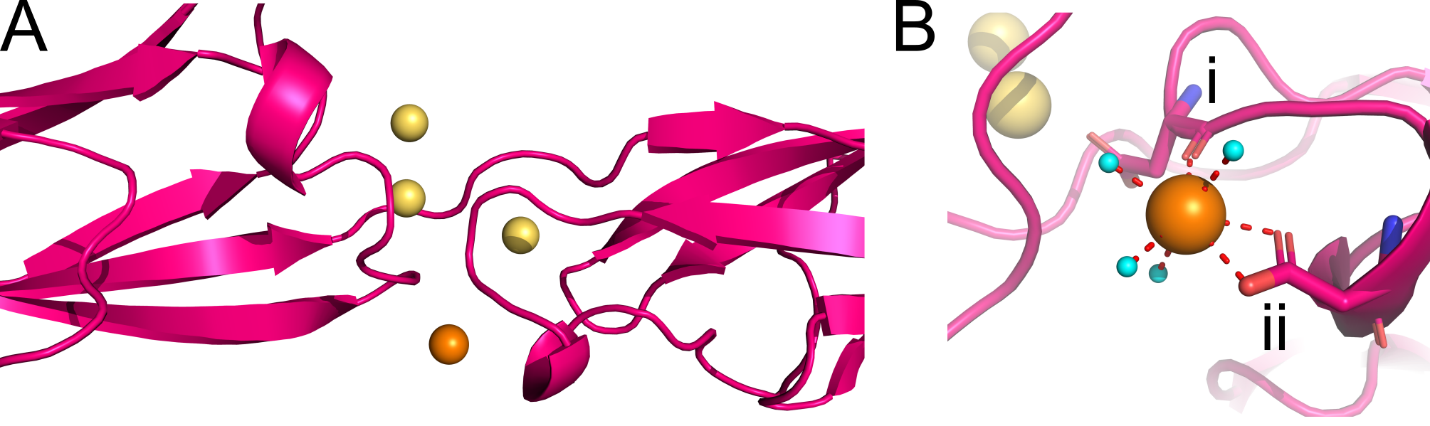


**Figure S2. Additional Ca^2+^ coordinated to the repeat interfaces in *Ah*Lap tetra-tandemer**. A) Aside from the consistent three Ca^2+^ (gold spheres) found between beta-sandwich repeats, occasional interfaces possess an additional ion (orange). B) The residues that coordinate the additional interface calcium ions are shown (i = Asp_backbone_374; ii = Asp_sidechain_377).


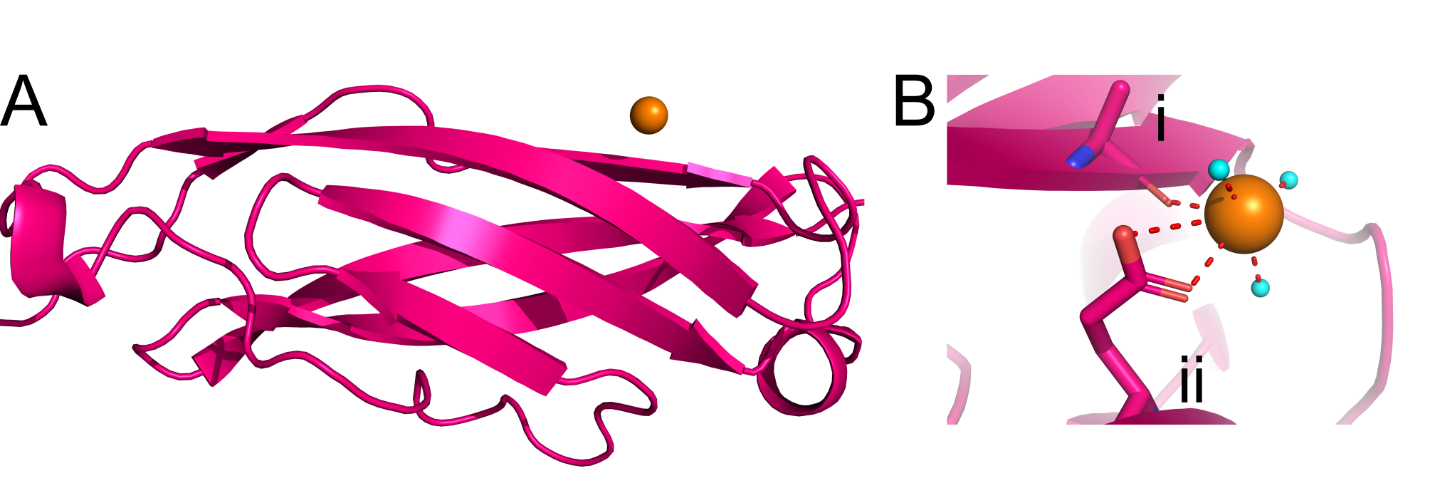


**Figure S3. Additional Ca^2+^ coordinated outside the interfaces in *Ah*Lap tetra-tandemer**. A) An additional Ca^2+^ (orange) is coordinated outside the repeat interfaces in only one instance. B) The residues that coordinate the additional calcium ion are shown (i = Ala_backbone_140; ii = Glu_sidechain_152).

**B) Supplementary Tables**

**Table S1. Deconvolution of a single beta-sandwich repeat from *Mp*IBP.**

|  | ***Mp*IBP Monomer** | |
| --- | --- | --- |
|  | 0 mM Ca^2+^ | 0.3 mM Ca^2+^ |
| Alpha helices | 3% | 12% |
| Beta strands | 9% | 38% |
| Turns & Unordered | 88% | 50% |
